# An Indication-Based Concept for Stepwise Spinal Orthosis in Low Back Pain According to the Current Literature

**DOI:** 10.3390/jcm11030510

**Published:** 2022-01-20

**Authors:** Franz Landauer, Klemens Trieb

**Affiliations:** 1 Department of Orthopaedic and Trauma Surgery, Paracelsus Medical University Salzburg, 5020 Salzburg, Austria; k.trieb@salk.at; 2 Computed Tomography Research Group, University of Applied Sciences Upper Austria, 4600 Wels, Austria

**Keywords:** back pain, low back pain, brace, spine orthoses, lumbar support, spine support

## Abstract

Background: The current literature is not conclusive for spinal orthosis treatment in low back pain. Therefore, two questions have to be answered: Does the current literature support the indication of spinal orthosis treatment in low back pain? Which treatment concept can be derived from the result? Method: The 30 highest-rated literature citations (PubMed: best match, 30 December 2021) dealing with low back pain and spine orthosis were included in the study. Excluded were all articles related to Kinesio Taping, scoliosis, physical exercise, or dealing with side effects and unrelated to treatment effect. Thus, the literature list refers only to “low back pain and spine orthoses”. These articles were analyzed according to the PRISMA criteria and divided according to “specific diagnosis”, when the cause of pain was explained (group A), or when “specific diagnosis is not given” (group B). The articles were also distinguished by the information about the orthosis. Articles with biomechanical information about the function of the orthoses were called “diagnosis-based orthosis” (group C). All other articles were part of the group “unspecific orthotic treatment” (group D). The results were compared to each other in terms of effectiveness. According to anatomical causes, a concept of orthosis selection depending on diagnosis of low back pain for clinical practice was developed. The risk of bias lies in the choice of the MESH terms. The synthesis of the results was a clinical treatment concept based on findings from the current literature. Results: The literature citations with 1749 patients and 2160 citations of literature were processed; 21 prospective clinical or biomechanical studies and 9 review articles were included. The combination of literature citations according to “specific diagnosis” (group A) and “diagnosis based orthosis” (group C) was very likely to lead to a therapeutic effect (seven articles). No positive effect could be found in four articles, all dealing with postoperative treatment. When “specific diagnosis is not given” (group B) and combined with “unspecific orthotic treatment” (group D), therapy remained without measurable effect (15 articles). An effect was described in four articles (three biomechanical studies and one postoperative study). In review articles, according to specific diagnosis, only one article dealt with fractures and another with stenosis. In all review articles where specific diagnosis was not given, no effect with spine orthoses could be found. Using this knowledge, we created a clinical treatment concept. The structure was based on diagnosis and standardized orthoses. According to pain location and pathology (muscle, intervertebral disc, bone, statics, postoperative) the orthoses were classified to anatomical extent and the mechanical limitation (bandage, bodice, corset, orthosis with shoulder straps and erecting orthosis). Conclusion: The effectiveness of spinal orthoses could not be deduced from the current literature. The most serious limitation was the inconsistency of the complaint and the imprecise designation of the orthoses. Interpretation: Articles with a precise allocation of the complaint and a description of the orthosis showed a positive effect. The treatment concept presented here is intended to provide a basis for answering the question concerning the effectiveness of spinal orthoses as an accompanying treatment option in low back pain.

## 1. Introduction

Spinal orthoses are used for spinal complaints in a very undifferentiated manner. This concerns the medical clarification as well as the selection of the aids. The international literature has referred to the clarification of a treatment algorithm.

The goal of orthotic care for spinal disorders is to reduce discomfort while activating the patient. This is achieved by segmental spinal stabilization and/or position correction. However, a targeted reduction of discomfort is only possible if the cause of the discomfort is known. This results in the requirement for a structured clinical examination, followed by imaging procedures.

A structured treatment concept is required for orthotic care, comparable to surgical planning. Undifferentiated indications bring orthotic treatment into disrepute, and the opinion of physicians regarding this form of treatment may be classified as negative. Nevertheless, the prescribing behavior is surprisingly generous in, for example, Austria. It is not the intention of this article to present this discrepancy. Rather, it aims to provide a guideline for structured care. The structure of this article is based on orthotics of the thoraco-lumbo-sacral spine.

The current literature is focused on lumbar complaints and thus on quickly available adaptable orthoses (adaptable device = industrial products).

This article attempts to reconcile the medical indications with the biomechanical criteria for orthotic fitting, and to address the following questions: does the current literature support the indication of spinal orthosis treatment in low back pain, and what treatment concept can be derived from the result? We hypothesized that if the cause of low back pain is not clarified and differentiated, no targeted effect may be expected from orthotic fitting.

## 2. Methods

The present article follows the current PRISMA criteria from 2020. This report comprises a literature review for which the current literature was first searched in PubMed on 30 October 2021 for the MESH terms “low back pain and spine orthoses”, then updated on 30 December 2021. Excluded were all articles related to Kinesio Taping, scoliosis, physical exercise programs and articles unrelated to treatment effect. Papers related only to a side effect were not included in the literature list. A general definition of low back pain and spine orthoses is given. Thus, the literature list refers only to “low back pain and spine orthoses” and resulted in 30 matches [1,2,3,4,5,6,7,8,9,10,11,12,13,14,15,16,17,18,19,20,21,22,23,24,25,26,27,28,29,30]. If the cause of the complaints was mentioned in an article, said article was assigned to group (A) with specific diagnosis.

Papers that did not provide a definite indication of the cause of pain were assigned to group (B), “specific diagnosis is not given”. Orthoses were differentiated in the same way. Articles with clear biomechanical information about the orthosis were called “diagnosis based orthosis” (group C). All other articles with only general biomechanical information were assigned to the group “unspecific orthotic treatment” (group D).

For the treatment concept, the next step was to create a structured differentiation of the orthoses. The differentiation was made in accordance with colloquial terms because a generally accepted definition or classification does not exist. In a further step, a standardization of the orthotic indication was created in accordance with the different causes of pain. This resulted in a clarification concept for the cause of pain and subsequently a functional, biomechanical treatment goal for the orthosis selection. This concept was summarized in tabular form.

The risk of bias lies in the choice of the MESH terms. The synthesis of the results yielded a clinical treatment concept based on findings from the current literature.

## 3. Results

**Figure 1:**
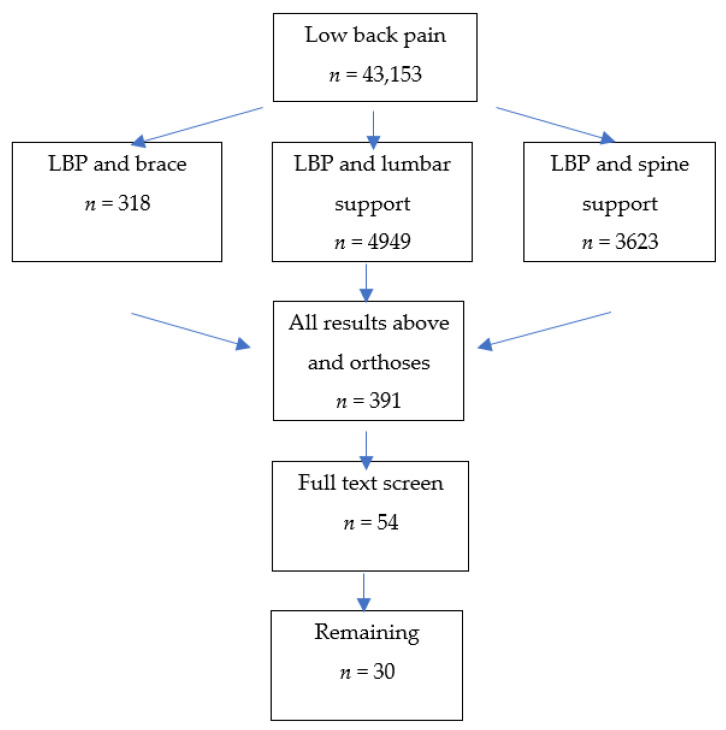
Flow diagram.

In the first search, 43,153 papers (“low back pain”) were found; when adding “brace”, 318 remained, 4949 for “spine support” and 3623 for “lumbar support”. For “orthoses”, 391 papers remained. The abstracts were searched for the exclusion criteria as described above. Thereafter, 54 papers remained for full-text screening, which resulted in full-text analysis of the 30 papers presented here with clinical relevance ([1,2,3,4,5,6,7,8,9,10,11,12,13,14,15,16,17,18,19,20,21,22,23,24,25,26,27,28,29,30], Figure 1).

Literature citations with 1749 patients and 2160 citations were processed. Ultimately, 21 prospective clinical or biomechanical studies and 9 review articles were included.

The following definitions of low back pain and spine orthoses were used:

Low back pain was defined as pain of musculoskeletal origin extending from the lowest rib to the gluteal fold that may at times extend as somatic referred pain into the thigh (above the knee). The definition of the North American Spine Society (978-1-929988-65-5) of an orthosis is as follows: A brace, splint, or other artificial external device serving to support the limbs or spine or to prevent or assist relative movement [13].

Many different terms are used for back orthoses (spinal orthoses, spine support, back supports, braces, bandage, girdle spine support, bodice, corsage, corset etc.).

This concerns the choice of words used, the indication for the orthotic fitting as well as its mode of action and design. A generally valid definition is lacking. There is only a general classification in ISO 8549-3:2020(en) (see Table 1, Table 2 and Table 3).

### 3.1. Overview of the Current Literature

The cause of the complaint must be diagnostically narrowed down.

The orthosis must be clearly defined in terms and function (Table 1).

Only when a differentiated cause of the complaint according to “specific diagnosis” was determined (group A) and the appropriately adapted “diagnosis based orthosis” was used (group C), were positive effects found in prospective studies ([2,5,9,22,28,30], Table 1), one retrospective study ([28], Table 1) and one review article ([10], Table 2).

No positive effect was prescribed in postoperative prospective studies ([20,23,25], Table 1) and one review article ([29], Table 2).

When no specific diagnosis is given (group B) and nonspecific orthotic treatment is administered (group D), no consistent result can be expected. The listed prospective studies ([1,6,8,11,14,17,18,19], Table 1) and review articles ([12,13,15,16,24,26,27], Table 2) demonstrated no positive effect. An effect was described in three biomechanical studies ([3,4,7], Table 1) and one postoperative study [21].

All meta-analyses and literature research reviewed did not provide medical or technical differentiation [1,6,8,11,12,13,14,15,16,17,18,19,24,26,27]. Thus, general orthotic designations such as “lumbar support” are used in the following. A general conclusion could not be found.

No review articles were identified where specific diagnosis was not given and orthosis with unspecific treatment was combined.

**Table 1:** Therapy effect in the literature for prospective studies and one retrospective study, depending on cause of complaint and orthosis.

Diagnosis		Orthosis
Group A According to specific diagnosis	With effect [2,5,9,22,30] (retrospective study 28)	Group C Diagnosis based Orthosis
	Without effect [20,23,25]	
Group B Specific diagnosis is not given	With effect [3,4,7,21]	Group D Orthosis as unspecific treatment
	Without effect [1,6,8,11,14,17,18,19]	

**Table 2:** Therapy effect in the literature for review articles depending on cause of complaint and orthosis.

Diagnosis		Orthosis
Group A According to specific diagnosis	With effect [10]	Group C Diagnosis based Orthosis
	Without effect [29]	
Group B Specific diagnosis is not given	With effect -	Group D Orthosis as unspecific treatment
	Without effect [12,13,15,16,24,26,27]	

### 3.2. Summary of the Literature and Whether It Supports the Indication of Spinal Orthosis Treatment in Low Back Pain

In the examined scientific literature we found a wide range of terms for orthoses. The terms did not give a clear indication of the orthosis used (orthosis, lumbar support, lumbar belt, brace, bandage, bodice, corset, pelvic orthosis, LSO (lumbosacral orthosis), low-profile exosuit, TLSO (thoraco-lumbar orthosis), hip orthosis, lumbar corset, bracing, back belt, rigid brace). Conversely, in biomechanical studies, clear descriptions could be found ([1,2,3,4,5,7,11], Table 1).

The medical descriptions of the complaint did not always indicate the cause of pain. Wordings were non-specific with regard to low back pain, back pain, etc. [6,8,14,17,18,19,22,27,28]. Within meta-analyses, review articles and an online survey, the causes of the pain were not differentiated [10,12,15,16,24,26,29]. In all cases of postoperative treatment, clear information about the clinical situation was given [20,21,23,25,29]. Only in one clinical study was clear information (MRI-Modic 1) given [28].

### 3.3. Differentiation of Spine Orthoses

In the following we make an effort to systematize the different terms used to describe orthoses and to link them to complaints and diagnostics.

Bandage (Soft orthosis with or without pads):

Protective, supporting bandage, soft/elastic (i.e., encompassing body parts with/without pads).

The indications for fitting are acute or chronic complaints diagnosed by clinical examination.

The mode of action is through a circular socket and force application via a pad.

**Figure 2:**
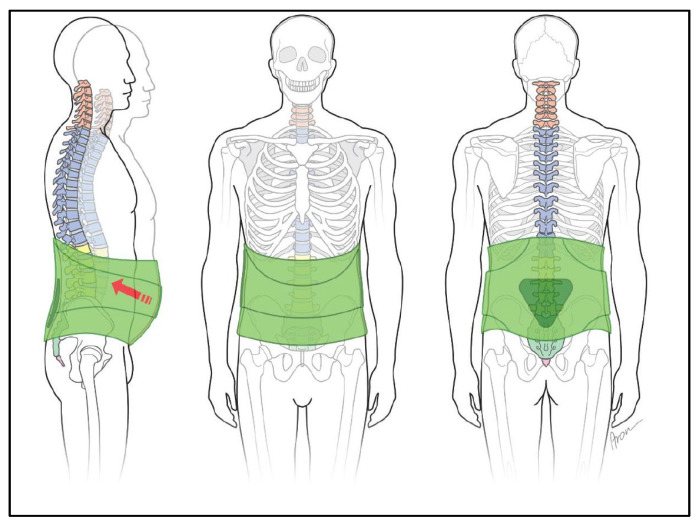
Bandage “Soft orthosis with or without pads”.

The diagnosis is considered to be mild, chronic or recurrent pain in the lumbar region (back support brace with dynamic pad, Figure 2).

Furthermore, spinal pain with moderate instability and lumbalgia are specified (lordosis/correction/bandage).

Bodice (girdle spine support, soft orthoses with struts, spine support, corsage):

Locally differentiated diagnoses are defined as indications for bodices. X-ray imaging is recommended for indication.

The mode of action is via stiffening elements that overlap and stabilize body regions. Delordosing of the lumbar spine is the major goal.

**Figure 3:**
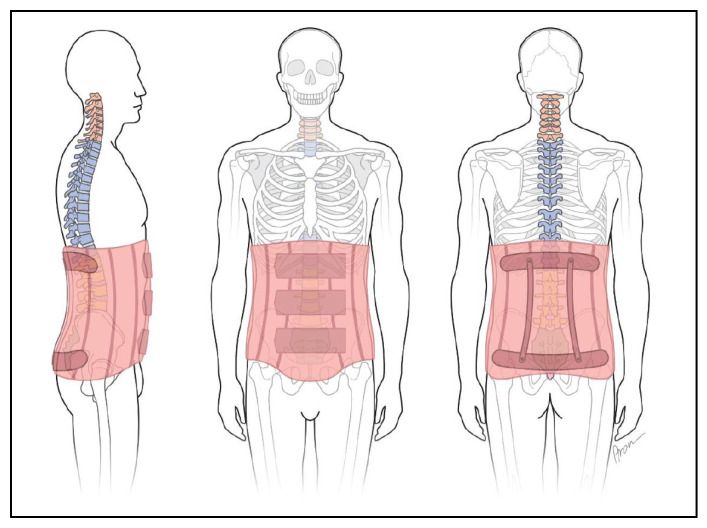
Bodice “soft orthosis with struts”.

The diagnosis of recurrent pain in the lumbar spine or at the thoraco-lumbar transition is the primary focus (e.g., lumbalgia, dorsalgia), postnucleotomy syndrome and for postoperative immobilization, but also instability, etc. (spine support with struts, Figure 3).

A distinction must be made between height differentiations with anatomical assignment as stretching only the lumbar spine (LSO) or including the thoracic spine (TLSO), etc.

Spine support with abdominal sling for delordosis and relief of the segments of the lumbar spine (in case of highly protruding abdomen, pregnancy, etc.).

Corset (soft orthoses with a rigid part to encompass the pelvis or a rigid orthoses):

Diagnosis of ailments that can be treated by correction and/or stabilization over the pelvic area form the indication for corsets.

Their mode of action is the correction in the sagittal and frontal planes and the limitation of rotation. This results in a degree of limitation that recommends slice imaging (CT or MRI) in most cases.

Segmental instabilities, spondylitis or spondylodiscitis, tumors and metastases, fractures without significant change in shape etc. are the most common indications.

**Figure 4:**
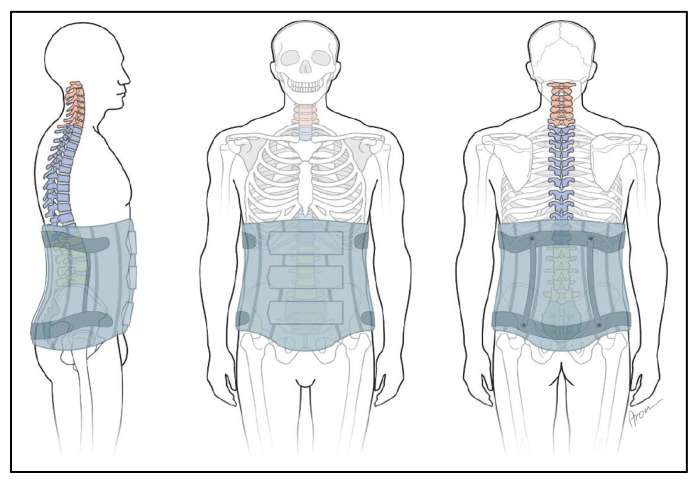
Corset “Soft orthoses with a rigid part encompass the pelvis”.

A special form of indication is a bridging corset with the possibility of gradual release of movement (e.g., as postoperative care; Figure 4). The corset becomes a bodice by removing the pelvic frame and a bandage by removing the stiffening elements. This gradually increases mobility.

Back brace with shoulder straps to erect the spine: dorsal struts with shoulder straps to erect the thoracic spine.

**Figure 5:**
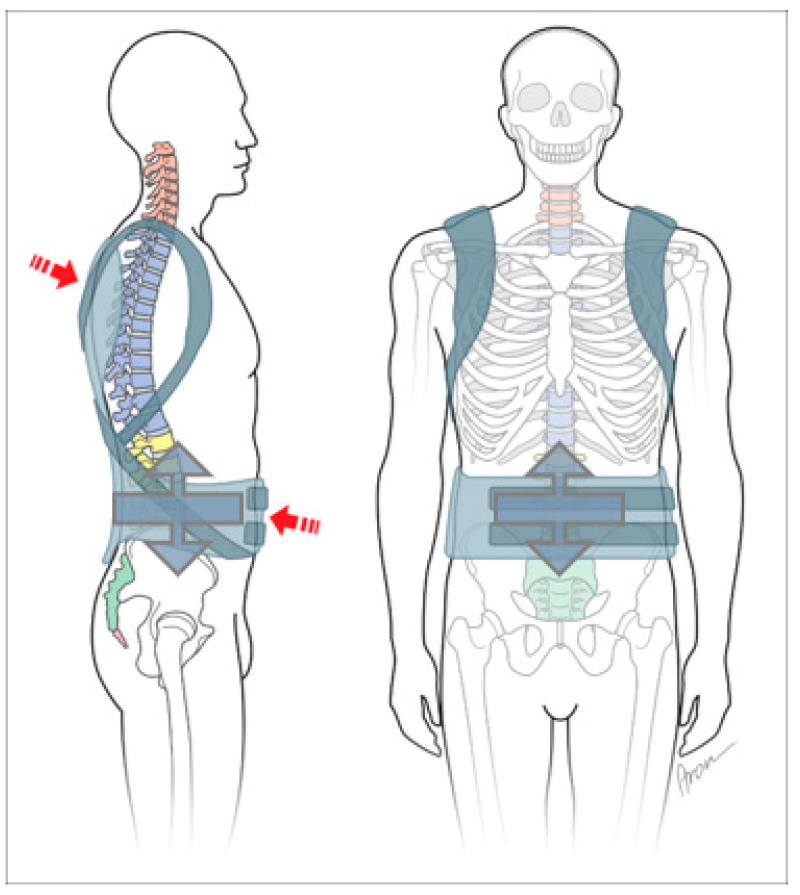
Back brace with shoulder straps to erect spine.

Symptomatic osteoporosis and hyperkyphosis in seniors are the most common indication for this treatment (Figure 5) [30].

**Figure 6:**
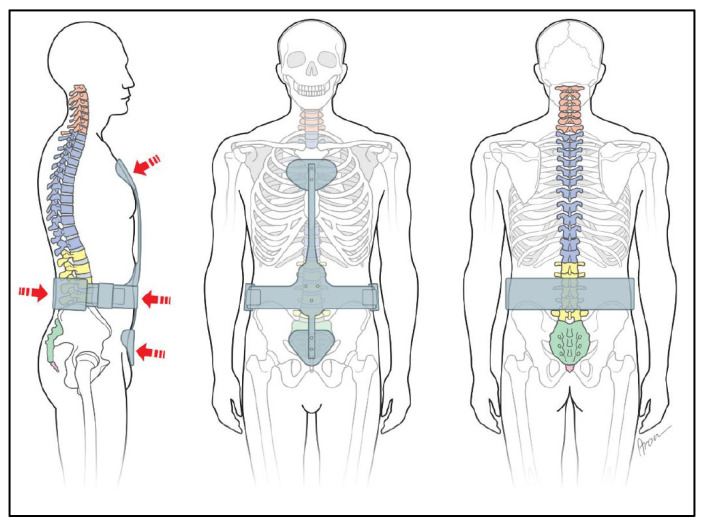
Spinal orthosis to relieve the spine.

Spinal orthosis which does not encompass the body to relieve spine: orthoses that do not encircle the trunk in a circular manner. Their aim is correction, especially in the sagittal plane. Thus, the indication for straightening the spine is in the primary focus. Vertebral fracture treatment without significant bony deformity forms the main indication (Figure 6).

Our goal was to derive a treatment concept on the basis of a delineation of the indications for different orthoses. 

According to the results of the literature review and classification of spinal orthosis, we made a standardization of indication. A clarification concept of the complaints followed by the orthosis selection depending on anatomical cause of the complaint was an element of the treatment concept.

In Table 3, pain localization is linked to pathology and orthosis selection is based on the anatomical localization and cause of the complaint. Table 3 gives an overview for a basic concept.

The result of a clinical examination is sufficient for the prescription of a bandage. Bandages can relieve pressure through the circular socket and, through the use of pads, provide targeted force application and thus pain relief (Figure 2).

Diagnoses derived from X-ray findings allow segmental assignment. Thus, a segmental force application or change in position of the spine is required to alleviate the symptoms. This correction can be achieved by a bodice (girdle spine support) (Figure 3).

A differentiated diagnosis by means of CT or MRI provides information that can be assigned to segmental anatomical structures. Only wearing a corset can provide the resulting stabilization or change of spine positioning (Figure 4).

In everyday use, there are overlaps between bodices and corsets.

A back brace with shoulder straps is indicated to erect the spine without affecting breathing. A fragile kyphotic spine, especially in elderly people, is the main indication (osteoporosis, metastasis, etc.) (Figure 5).

Spine supports which do not encompass the body are a possible treatment of vertebral body fractures without loss of stability, and support the healing conservatively (according to AO classifications A0 and A1) (Figure 6).

For prevention, wearables have become increasingly available as a training device for postoperative treatment and to avoid discomfort. The line between medical treatment and sports equipment is becoming increasingly blurred.

**Table 3:** Standardization of indication and therapy goals.

	Diagnosis		Orthosis		
Summary	Back pain		Spinal orthosis		
Local distribution	Cervical syndrome Dorsalgia Lumbalgia Sacralgia		Cervical orthosis (CO) Thoracic orthosis (TO) Lumbo-sacral orthosis (LSO) Sacro-iliac orthosis (SIO)		
	Combination		Thoraco-lumbar-sacral orthosis (TLSO) Cervico-thoraco-lumbo-sacral orthosis (CTLSO)		
Pathology	Diagnosis	Therapy	Mode of action	Product	
Muscle	Lumbalgia	Relief	Targeted force application via pads	Bandage	
Disc	Lumbalgia Lumboischialgia Instability	Stabilization Positioning	Delordosization through dorsal bridging	Bodice Corset Back brace Spinal orthosis	Depending on the necessary stability
Bone	Spondylarthrosis Baastrup	Position change	Delordosis		
	Osteochondrosis Osteoporosis	Stabilization			
	Fracture Metastases	Stabilization Prevention			
Static	Hyperkyphosis Hyperlordosis Scoliosis	Position correction			
Postoperative	Post segmental fusion	Stabilization Protect the adjacent segment Rapid mobilization	Local relief	Soft orthosis with struts	
Training device	Long-term treatment or prevention	Activation	Stimulators Assistive products	Wearables	

## 4. Discussion

This manuscript demonstrates missing links between low back pain and orthotic fitting. As a result of these findings, we developed a concept for orthotic selection depending on the pathological source. This could be considered a step towards improving the accompanying treatment options and therapeutic accuracy.

Spinal orthoses can limit range of motion, and can stabilize or reposition spinal segments. In the case of obesity or during pregnancy, the circular design can relieve the spine through targeted application of ventral forces. Other local effects are described, but not well studied. For temporary orthotic treatment, the effects must be consistent with the cause of the complaint.

One prospective study did not show any effectiveness of lumbar supports in 28 assembly-line workers, with respect to low back functionality and disability [1]. In contrast, another reported positive effects of wearing a lumbar belt in workers [4]. An in vitro biomechanical study investigating the effects on posterior pelvis kinematics reported an altered lumbosacral transition and increased movement in the sacroiliac joint by pelvic orthosis [5].

Two prospective reports dealt with postoperative bracing including 119 patients. No indication for postoperative bracing regarding pain relief or quality of life was observed [22,23]. The same was true for 96 patients with conservatively treated thoraco-lumbar burst fractures [9] or following single-level lumbar discectomy in 54 patients [20].

Regarding low back pain, different study protocols reported a positive effect of lumbar orthosis in 115 patients [2,6,18,21]. In contrast, four different randomized studies did not provide any pain relief after six months observation of 266 patients [8,14,17,30]. No positive effect of wearing a lumbar orthosis on muscle thickness measured by ultrasound was reported in 44 patients [19]. Reduced back muscle fatigue was reported in six healthy participants after wearing a low-profile elastic exosuit [7].

Here we only individually discuss those contributions that describe no effect despite differentiated cause of pain and differentiated orthotic fitting. In total, 30 studies were identified, of which 20 were prospective studies (Table 1) and nine were reviews or meta-analyses (Table 2). The remaining paper was a retrospective analysis of treating chronic lumbar back pain with a rigid lumbar brace [28]. The literature selection process is presented in the flow diagram in Figure 6.

Zoia et al. investigated postoperative orthotic fitting after monosegmental disc surgery. Their highly structured study concluded that after monosegmental disc surgery, the short-term and mid-term outcomes displayed no difference to orthosis-free care [20].

It should be noted that all patients displayed monoradicular lumboischialgia and were surgically treated by only two surgeons using microscopes. The corresponding pain reduction did not suggest an effect of additional orthoses, since a sufficient postoperative pain treatment would be expected. The question about a possible reduction of pain medication, recurrence frequency or longer-term instability has not been answered.

Fujiwara et al. reported that orthotic fitting after PLIF (posterior lumbar interbody fusion) resulted in no benefit compared to orthotic-free fitting. Severe osteoporosis was mentioned as an exception [23].

Neither a benefit nor a disadvantage in terms of complaints was recorded in the control period of 3 months. In this case, it was also true that drug therapy was not answered. A long-term effect on follow-up degeneration was not discussed.

Orthotic fitting after posterior instrumented fusion did not improve quality of life or complaints, as reported by Soliman. The number of complications and reoperations in the brace group (7 out of 25) and in the control group (5 out of 18) must be seen as a limitation on the outcome [25].

Disc surgery, or segmental fusion, should not require additional external stabilization. However, this only applies to the surgically treated segment. The protection of the adjacent segment and thus the reduction of connecting degenerations cannot be derived from this.

It must also be considered that a sufficient postoperative medicinal pain treatment excludes the discomfort as a measure for an indication of the orthotic fitting.

From this perspective, local wound treatment, reduction of complications and, in the long term, prevention of follow-up degeneration should be cited as measures of treatment success with spine orthoses.

In a Cochrane Database of Systematic Reviews from 2016, the “Surgical versus non-surgical treatment for lumbar spinal stenosis” was treated. The authors concluded that they had very little confidence to confirm whether surgical treatment or a conservative approach is better for lumbar spinal stenosis, and they could not provide any new recommendations to guide clinical practice [29].

The results of the current literature are presented in a structured manner. Overall, there are no general statements for or against treatment with orthoses in low back pain.

The simple systematic structuring of orthoses forms the basis for finding promising treatments, but also forms a basis for contraindications.

Strict scientific statements cannot be provided. The structure of the orthoses is influenced by clinical application, since neither the nomenclature for orthoses nor a recommendation for indications is currently available.

This manuscript highlights the weakness of the current indications for orthotic provision in low back pain.

Accordingly, we can conclude that the current literature gives no recommendations to guide clinical practice.

The effectiveness of spine orthoses cannot be deduced from the current literature. The most serious limitation is the inconsistency of the complaints and orthoses. Furthermore, the imprecise designation of the orthoses is an additional limitation.

## 5. Conclusions

The effectiveness of spinal orthoses cannot be determined on the basis of the current literature. A major limitation is the lack of standardized nomenclature.

The lack of differentiation of the causes of pain is another weakness in many scientific papers. This limitation cannot be overcome by statistical methods or meta-analyses.

Additionally, in the case of postoperative treatments, pain reduction cannot be applied as a measure of therapeutic success in the presence of sufficient medicinal pain management.

The categorization of spinal orthoses demonstrated in this manuscript should be an impetus for further efforts to standardize products. These suggestions can provide the basis for answering the question of the effectiveness of spinal orthoses in conjunction with a differentiated cause of complaints. Spinal orthoses are an additional treatment option in limited indication of medication or surgery.

We found that articles with a precise allocation of the complaint and a description of the orthosis showed a positive effect. The treatment concept presented here is intended to provide a basis for answering the question concerning the effectiveness of spinal orthoses as an accompanying treatment option.

## Data Availability

The study did not report any data.
